# Biomechanical impact of severe genu valgum (18°) on the lateral meniscus: a finite element stress analysis

**DOI:** 10.3389/fbioe.2026.1860122

**Published:** 2026-08-20

**Authors:** Miguel Angel García-Laguna, Guillermo Urriolagoitia-Sosa, Beatriz Romero-Ángeles, Miguel Martinez-Mondragon, Jorge Alberto Gomez-Niebla, Luis Itzcoatl Lugo-Chacon, Alfonso Trejo-Enríquez, Jonathan Mireles-Hernández, Francisco Javier Gallegos-Funes, Erick Velazquez-Lozada

**Affiliations:** Instituto Politécnico Nacional, Escuela Superior de Ingeniería Mecánica y Eléctrica, Sección de Estudios de Posgrado e Investigación, Unidad Profesional Adolfo López Mateos Zacatenco, Ciudad de México, Mexico

**Keywords:** finite element analysis, genu valgum, knee biomechanics, meniscus, osteoarthritis

## Abstract

Genu valgum is a deformity that alters load transmission in the knee. While the effects of mild deviations are known, the specific quantitative impact of severe deformities (>15°) on the lateral meniscus remains understudied. This research aims to quantify the stress distribution in a severe 18° valgus deformity compared to a healthy morphology. A comparative Finite Element Analysis (FEA) was conducted. 3D models were reconstructed from segmented DICOM images using MIMICS and 3MATIC software. A static vertical load of 850 N, simulating a bipedal stance, was applied to both a healthy model and a pathological model with an 18° axial deviation. The simulation used a high-order 20-node quadratic mesh to ensure accurate capture of stress gradients. The healthy knee exhibited a maximum Von Mises stress of 2.76 MPa, distributed homogeneously. In contrast, the pathological model showed a critical stress concentration of 17.54 MPa in the lateral meniscus. This represents a 535% increase, demonstrating a non-linear exponential rise in stress compared to milder deformities reported in the literature. An 18° valgus deformity subjects the lateral meniscus to stress levels exceeding physiological fatigue limits, confirming the mechanism for the rapid onset of lateral unicompartmental osteoarthritis. These findings suggest that conservative therapies are biomechanically insufficient for such degrees of deformity, validating the necessity for corrective osteotomy and the use of FEA as a preoperative planning tool.

## 1 Introduction

**FIGURE 1:**
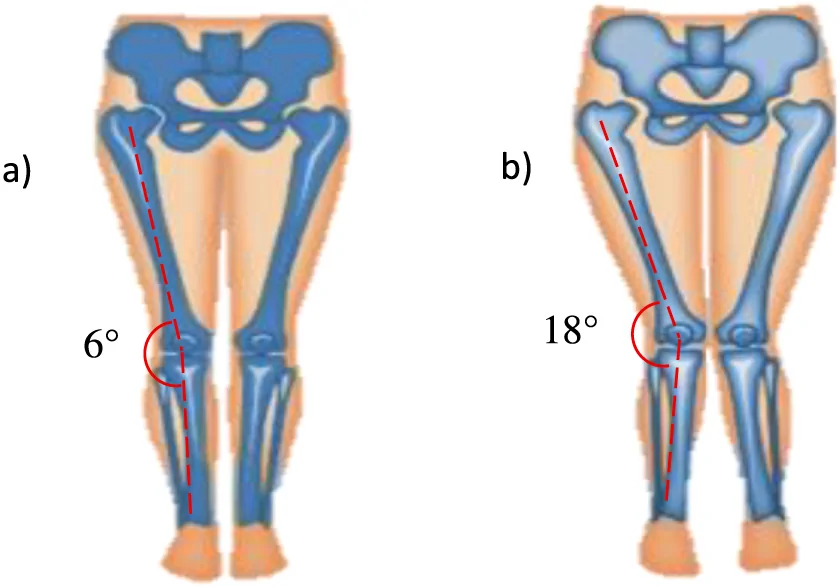
Knee configuration. **(a)** Healthy knee. **(b)** Knee with genu valgum pathology.

The knee is a critical element of the lower limb kinematic chain, providing the necessary stability and mobility for daily activities such as walking, running, and load-bearing. Due to its weight-bearing function and the repetitive nature of these movements, this joint is highly susceptible to injuries resulting from biomechanically inefficient postures, trauma, or hereditary factors that alter its mechanical axis. Prominent among these alterations is genu valgum, a pathology defined by the mechanical displacement of the tibiofemoral ax-is in the coronal plane (Figure 1).

Physiologically, during childhood development, the tibiofemoral angle tends to correct naturally, stabilizing between 5° and 7°. However, when this angulation exceeds 8°, it is considered a pathological condition. This deformity generates asymmetric loading, concentrating stress in the lateral compartment of the knee, triggering a cascade of biomechanical deterioration ranging from aesthetic issues and gait alterations to joint instability and accelerated cartilage and meniscal wear. If left uncontrolled, this condition evolves into severe cases of unicompartmental osteoarthritis, requiring complex interventions.

**FIGURE 2:**
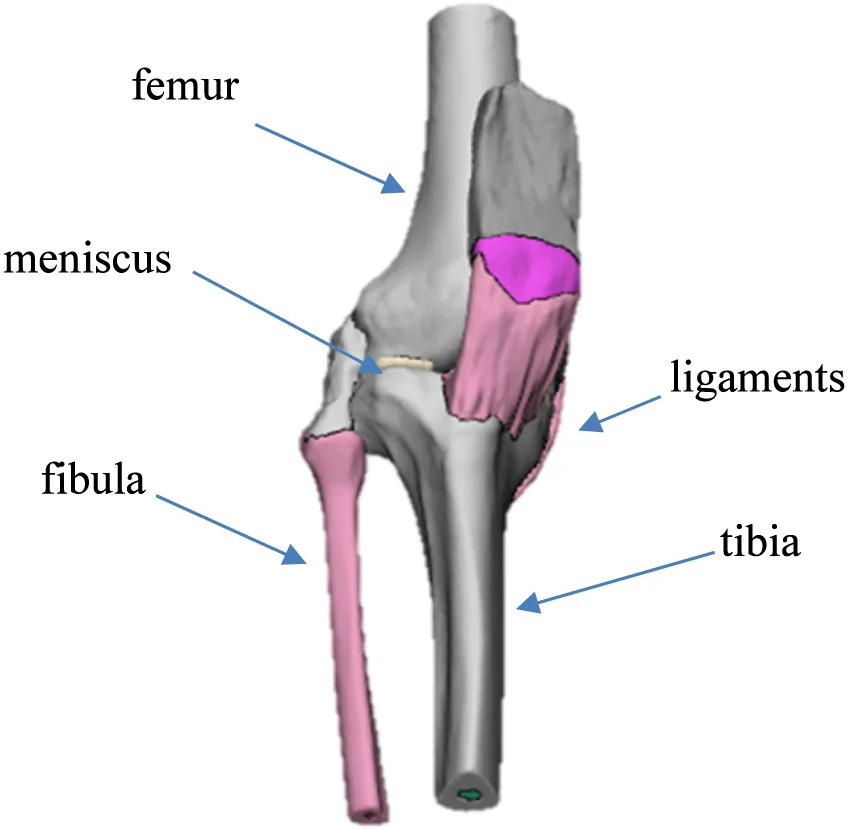
CAD knee model.

The biomechanics of these deviations have been studied using various methodologies. Previous research, such as that by Van Gheluwe, has analyzed the effects of simulated genu valgum on ground reaction forces and subtalar joint function during gait. Likewise, the use of Finite Element Analysis (FEA) has proven effective in modeling the behavior of knee prostheses under different loading conditions, as demonstrated by Kumbhalkar (Viladot-Voegeli, 2001; John et al., 2013). Furthermore, recent advancements in computational biomechanics have increasingly relied on patient-specific FEA models to predict localized wear patterns, evaluate complex knee deformities, and optimize surgical planning, reaffirming the clinical utility of these numerical methods (Zhang et al., 2025; Peitola et al., 2025; Abdollahpour et al., 2025). The resulting assembled CAD model, including the distinct anatomical regions, is presented in Figure 2. Despite the availability of conservative treatments based on physical therapy, clinical efficacy is often compromised by inconsistent patient adherence, frequently due to the incompatibility of therapies with sports practice or the underestimation of the pathology’s progressive severity.

**FIGURE 3:**
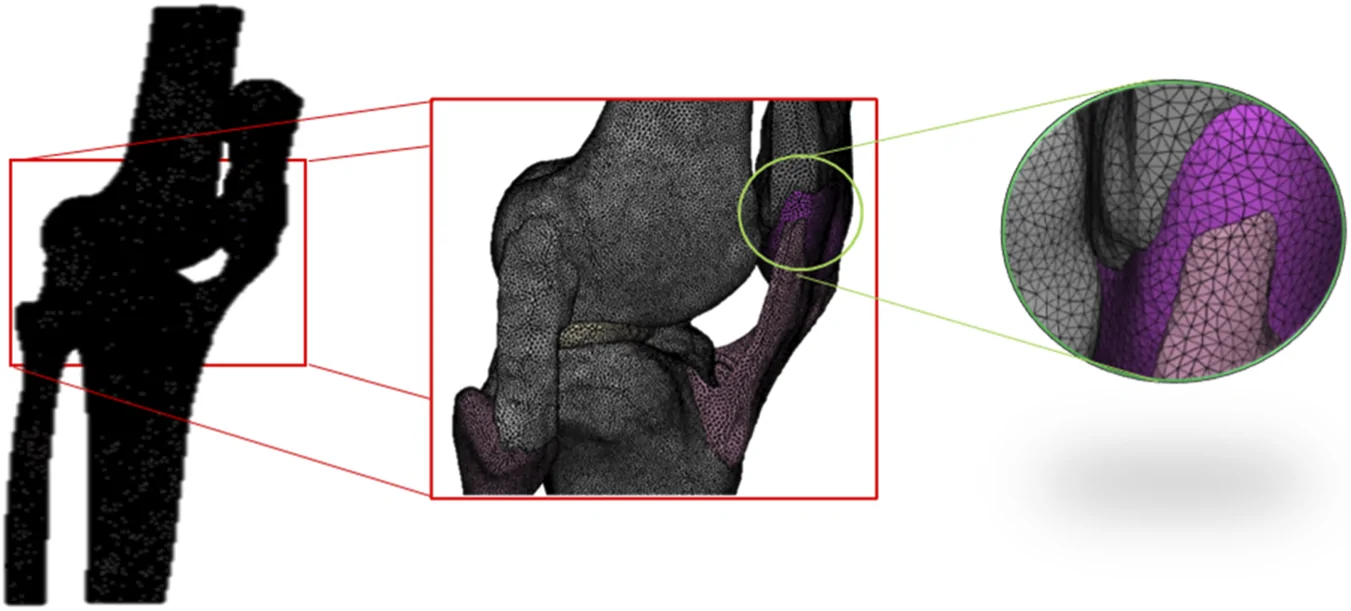
Discretized biomodel.

Given the need to precisely quantify the impact of these deviations to justify timely interventions, this study proposes a comparative computational simulation approach. The main objective is to analyze the biomechanical behavior of the knee under two conditions: a healthy morphology and a pathological model with an 18° genu valgum deformity, and to evaluate differences in stress and strain distribution, providing quantitative data to as-sist in medical planning and the design of corrective treatments. These critical meshing details are comprehensively illustrated in Figure 3.

## 2 Methodology to produce a biomodel for numerical analysis

**FIGURE 4:**
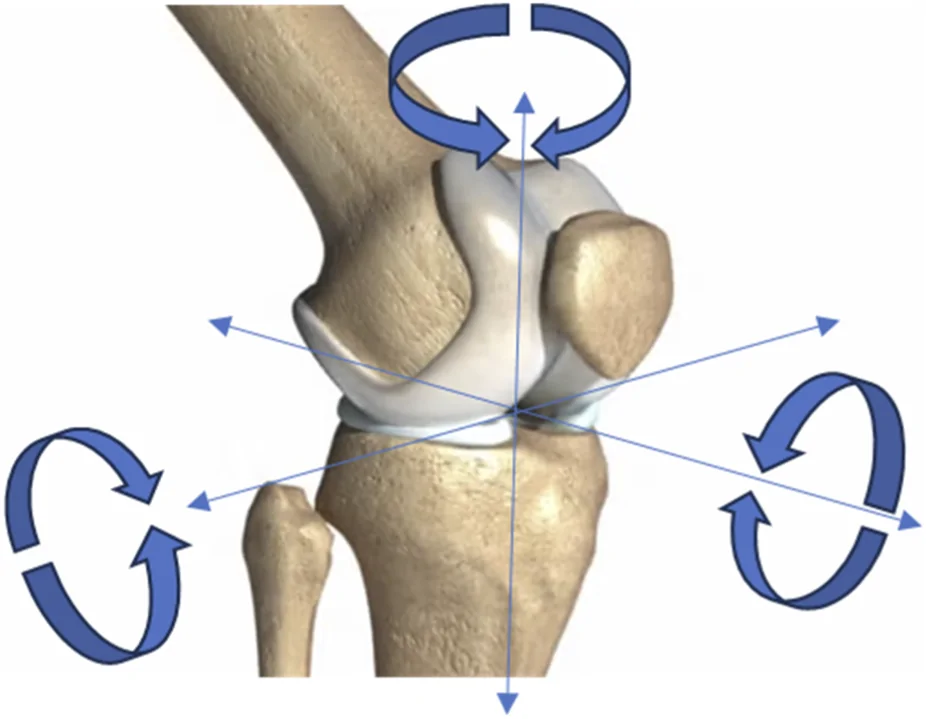
Knee movements.

Knee kinematics presents a biomechanical architecture that exceeds the function of a simple hinge, characterized by six degrees of freedom. As illustrated in Figure 4 this complexity arises from the simultaneous interaction of three translation vectors (anteroposterior, mediolateral, and proximodistal) and three rotational movements (flexion-extension, internal-external rotation, and varus-valgus deviation).

**FIGURE 5:**
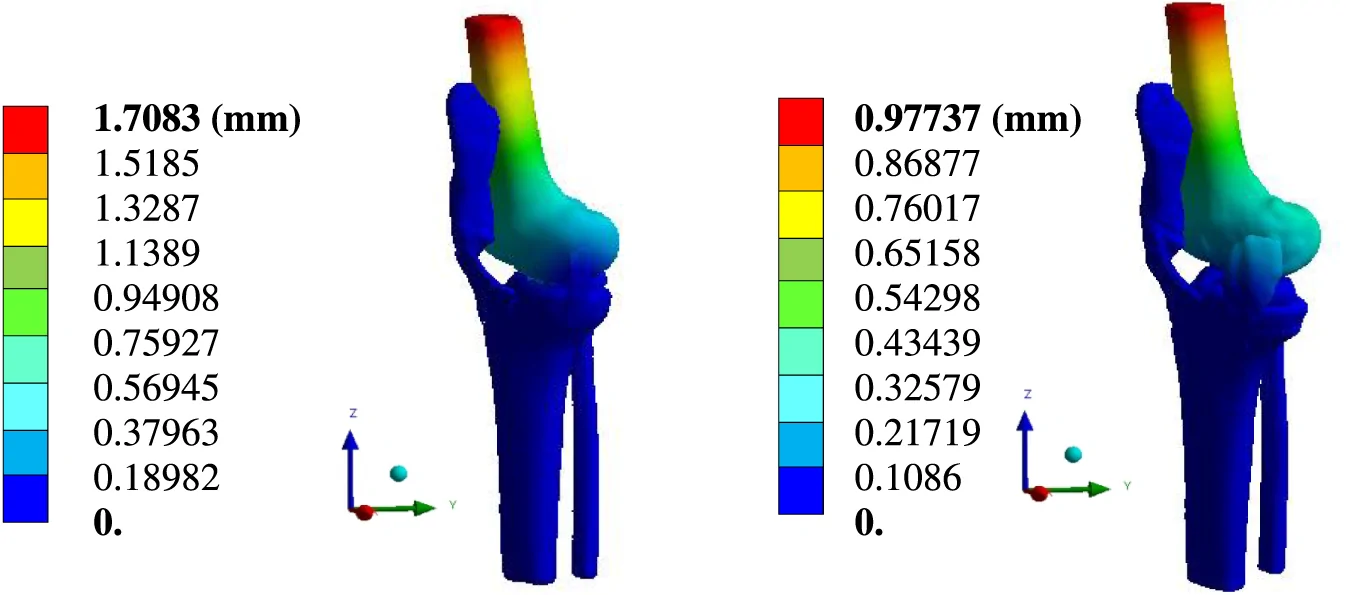
Total displacement.

**FIGURE 6:**
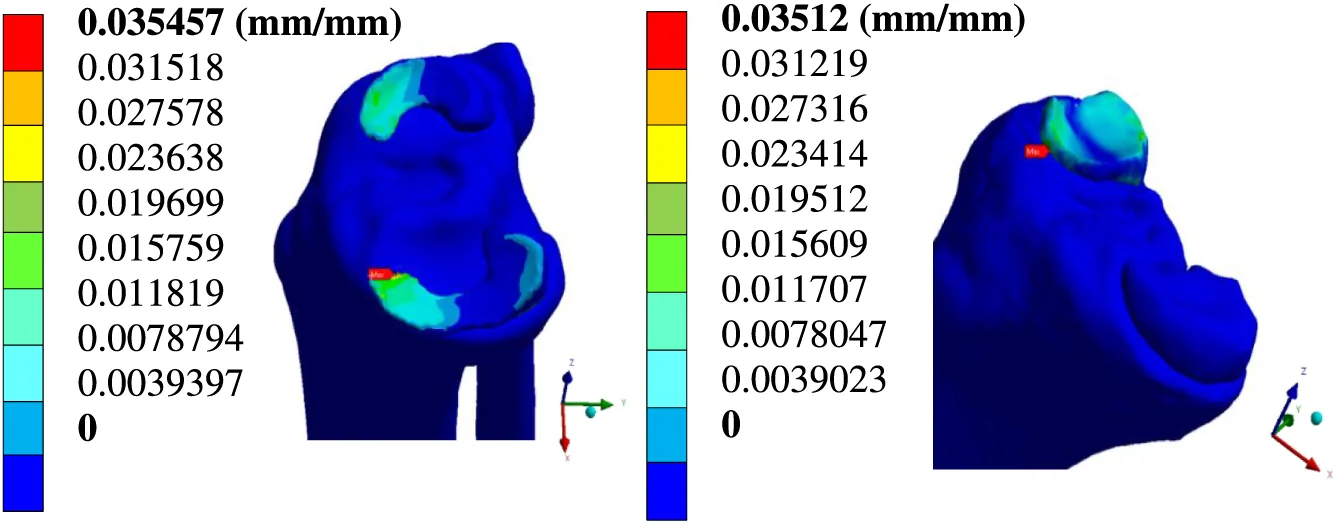
Deformation unit total.

**FIGURE 7:**
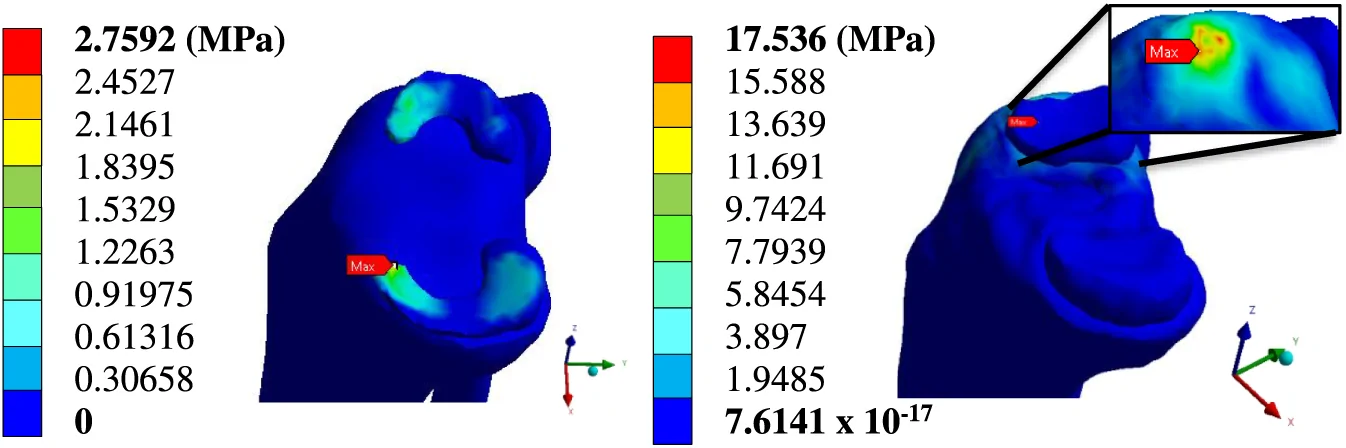
Von mises stress.

Figure 5 illustrates the total displacement field. Subsequently, Figure 6 depicts the equivalent elastic strain. Finally, Figure 7 shows the Von Mises Equivalent Stress distribution.

Given this anatomical complexity and the ethical and practical limitations of *in vivo* instrumentation to measure internal stresses in a multidirectional kinematic environment, this study adopted an approach based on the Finite Element Method (FEM) using ANSYS® software. This numerical technique was selected for its ability to solve complex differential equations governing the structural behavior of biological tissues, enabling both linear and nonlinear analyses with high precision. The computational model serves as a critical non-invasive tool for quantifying stress and strain distributions in the lower limb kinematic chain, overcoming the limitations of direct experimentation on living subjects and providing a reliable approximation of joint mechanics under physiological loads.

**FIGURE 8:**
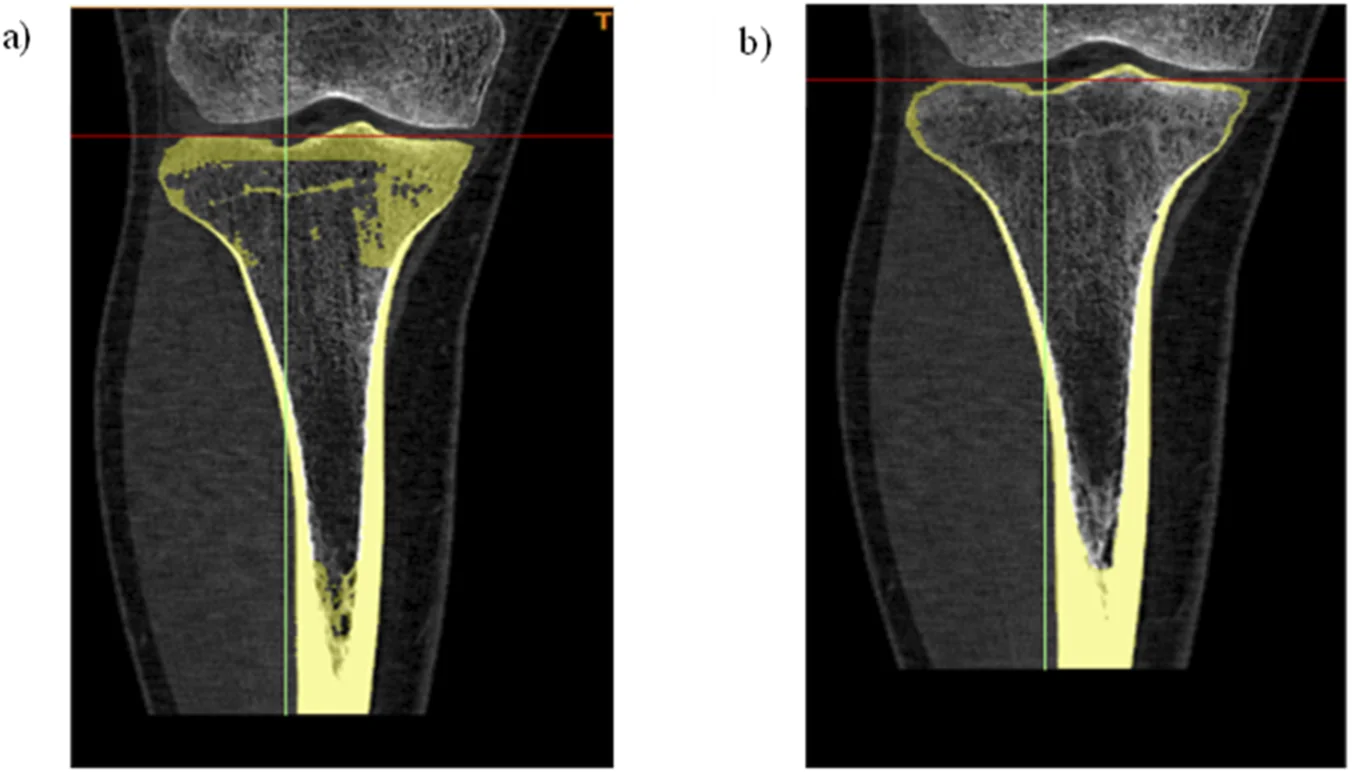
MRI. **(a)** Image obtained by tomography. **(b)** Image correction.

The anatomical re construction process employed MIMICS® software, beginning with the acquisition of high-resolution bioimages in DICOM (Digital Imaging and Communications in Medicine) format, obtained through Magnetic Resonance Imaging (MRI) or Computed Tomography (CT). These volumetric images were processed using digital segmentation algorithms to isolate the bone structures and soft tissues of interest (femur, tibia, menisci), differentiating tissues according to their grayscale density (Figure 8).

The point clouds generated during segmentation were exported to 3-matic®, a Computer Aided Design (CAD) software, for geometric post-processing. In this phase, reverse engineering techniques were applied to ensure the fidelity of the biomodel, including:

Mesh repair: Correction of discontinuities, hole filling, and reconstruction of missing surfaces.

Surface smoothing: Elimination of defects and digital noise to prevent artificial stress concentrations during simulation.

Solidification: Conversion of the surface mesh into solid volumes suitable for discretization.

This methodological workflow enabled the generation of accurate virtual geometry, thereby optimizing the study’s cost-effectiveness by substituting costly physical prototypes with iterative digital simulations.

## 3 Numerical analysis

Following geometric optimization, the biomodel was imported into the simulation environment for discretization. The continuous domain was subdivided using an adaptive discretization strategy, governed by the topological complexity of the articular surfaces. To ensure fidelity in representing the condylar curvatures and to accurately capture stress gradients, high-order volumetric elements with quadratic shape functions (20 nodes per element) were selected. This configuration enables parabolic interpolation of displacements, significantly reducing discretization error and mitigating the artificial stiffening effects inherent to complex geometries.

Once the discretization was defined, the process proceeded to the assignment of the constitutive equations governing the mechanical behavior of the tissues. A linear elastic and isotropic behavior were assumed for both bone structures and soft components, assigning specific moduli of elasticity (Young’s Modulus) and Poisson’s ratios for each volume (cortical bone, trabecular bone, menisci, etc.), as detailed in Table 1. These properties were consistently applied in both the control (health) model and the pathological model to ensure the study’s comparative validity (Maya-Anaya et al., 2023).

**TABLE 1:** Mechanical properties.

Element	Young’s module (MPa)	Poisson’s relationship
Cortical bone	18,000	0.3
Trabecular bone	75.5	0.3
Anterior cruciate ligament	64	0.4
Posterior cruciate ligament	67	0.4
Lateral ligament	61	0.4
Meniscus	55	0.3
Tendon	338	0.45

**FIGURE 9:**
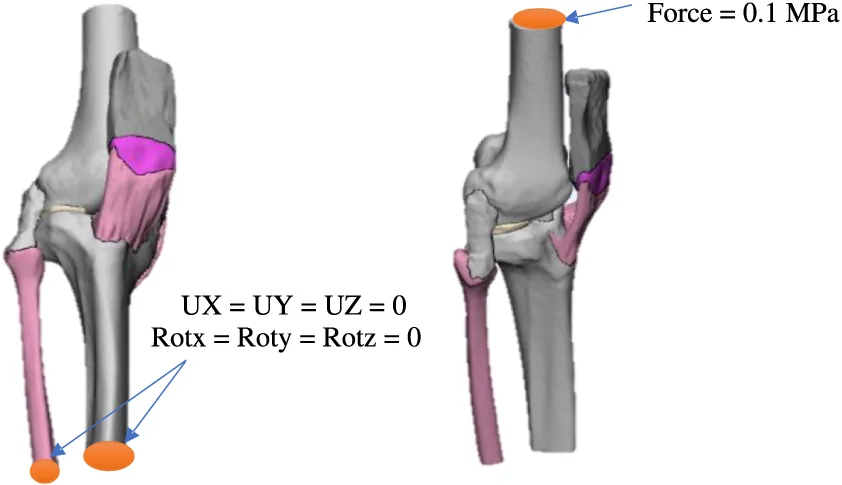
Force and movement restrictions.

To simulate the biomechanical environment of a static bipedal stance, strict boundary conditions were established within a multibody interaction analysis. All degrees of freedom (UX = UY = UZ = 0; ROTx = ROTy = ROTz = 0) were constrained at the distal tibial epiphysis to simulate a distal fixed support. Simultaneously, an axial load of 850 N was applied to the proximal femoral surface, replicating the body weight reaction on the joint. Accurate definitions of these constraints and load vectors, as illustrated in Figure 9, are critical for the convergence of the global stiffness matrix and the physiological relevance of the resulting results (Peña et al., 2006).

**FIGURE 10:**
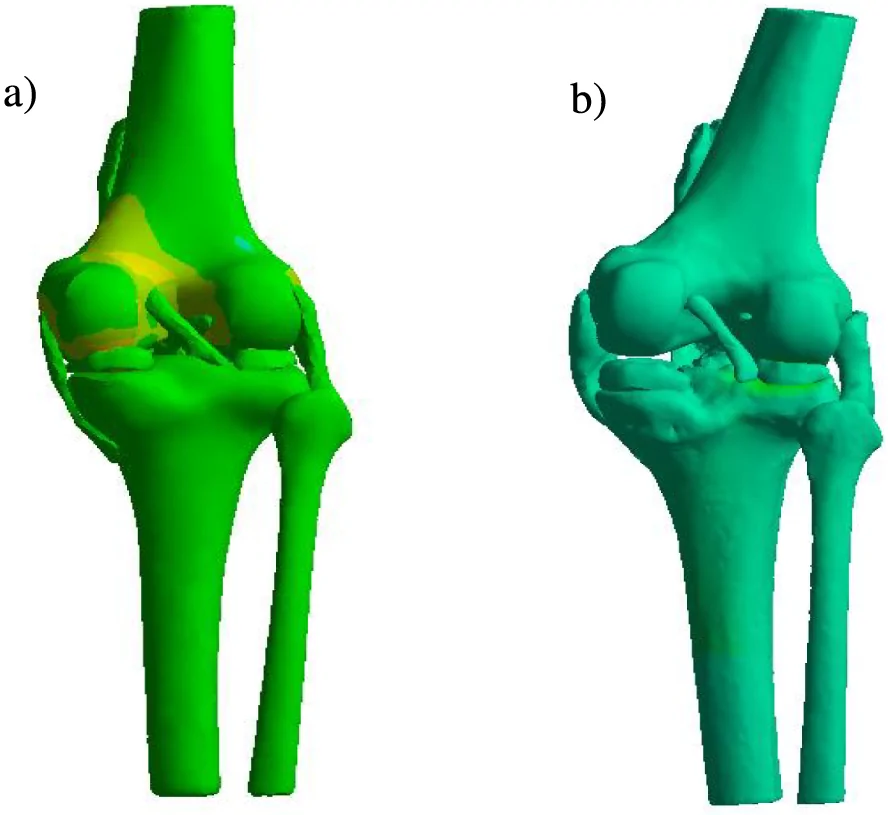
**(a)** Healthy knee. **(b)** Knee with pathology.

The simulation protocol was standardized for both scenarios. Constitutive properties, boundary conditions, and applied loads were identical for the control (healthy) model and the pathological (genu valgum) model, the comparative configuration of which is detailed in Figure 10.

It is imperative to highlight that the reliability of the results obtained via the Finite Element Method (FEM) is intrinsically dependent on the methodological rigor applied during the pre-processing phases (segmentation, reconstruction, and meshing). In contemporary computational biomechanics, numerical validation serves not only as a predictive tool but also as an operational efficiency strategy; implementing these simulations allows the substitution of physical prototype manufacturing iterations and destructive mechanical testing, substantially reducing development costs and optimizing analysis times without compromising biological precision.

## 4 Results

The biomechanical behavior of the studied models was evaluated by post-processing the finite element analysis results. The results are presented as color-coded stress fields, allowing a direct visual correlation between anatomical geometry and the calculated physical magnitudes.

This graphical representation facilitates the identification of critical zones using a normalized color scale, where red tones denote the highest magnitude peaks indicative of stress concentrations or critical displacements, while blue tones represent the lowest values. The analysis focused on three fundamental indicators for the joint’s structural integrity.

- Von Mises Equivalent Stress: To evaluate the yield criterion.
- Total Resultant Displacement: To quantify the system’s global stiffness.
- Maximum and Minimum Principal Stresses.

## 5 Discussion and conclusions

The present research quantified the biomechanical impact of severe genu valgum deformity (18°) on the knee joint. The most critical finding was the drastic increase in Von Mises Equivalent Stress on the lateral compartment. While the healthy morphology model exhibited a maximum stress of 2.76 MPa distributed homogeneously, the pathological model concentrated a load of 17.54 MPa on the lateral meniscus. This represents a 535% increase in local stress magnitude, a disparity that confirms the hypothesis of accelerated deterioration in patients with mechanical axis deviations greater than 10°.

The values for the healthy knee (2.76 MPa under a static load of 850 N) are in good agreement with previous finite-element validation studies. For instance (Peña et al., 2006), reported meniscal contact pressures of 2.5–3.5 MPa under similar bipedal loading conditions, validating the fidelity of the boundary conditions and constitutive properties assigned in Table 1.

However, the discrepancy observed in the genu valgum model is significant when contrasted with moderate deformities. Studies such as those by (Yang et al., 2010), which analyzed valgum deviations of 5°–10°, reported stress increases of 50%–100% in the lateral compartment. Our study, which evaluates a severe deformity of 18°, evidences a non-linear behavior in which stress not only doubles but sextuples. This suggests the existence of a critical angular deformity threshold, beyond which meniscal compensation mechanisms collapse, transferring destructive loads directly to the subchondral bone.

The peak value of 17.54 MPa exceeds the physiological fatigue limit of articular cartilage and dangerously approaches the failure limit of meniscal tissue reported in biomaterials literature by (Adouni, M. et al., 2012). While most studies focus on mild deformities of 5°, there is a lack of quantitative data on meniscal behavior in severe deformities greater than 15° prior to total collapse. Therefore, this biomechanical finding provides a quantitative explanation for the clinical observation described by (Van-Gheluwe, 2005), who associates genu valgum with joint instability and progressive pain. The stress concentration in a reduced area of the lateral compartment explains the pathogenesis of early lateral unicompartmental osteoarthritis. Clinically, these results justify the need for corrective surgical interventions, such as distal femoral osteotomies, in patients with angles greater than 15°, as conservative therapies described in the introduction would be biomechanically insufficient to mitigate the stresses of this magnitude.

While the comparative analysis provided conclusive data on load distribution, several methodological simplifications must be explicitly acknowledged as limitations that affect the absolute physiological realism of the model. First, the assumption of linear, elastic, and isotropic material properties for soft tissues particularly the menisci and ligaments represent a simplification of their true hyperelastic, anisotropic, and viscoelastic nature. Second, the simulation relies on a single static axial load (850 N) representing a bipedal stance, omitting dynamic loading conditions such as the complete gait cycle. Furthermore, the model does not include the stabilizing and counteracting active muscle forces (e.g., quadriceps and hamstrings) nor complex joint kinematics. Finally, while the stress baseline of the healthy model correlates with existing validated literature, the pathological model with an 18° deviation lacks direct experimental validation due to the ethical and practical impossibilities of vivo measurement under such severe structural compromise. Nevertheless, by isolating the geometric variable and applying consistent boundary conditions to both models, this study provides a highly controlled, conservative worst-case biomechanical scenario. While absolute stress values might vary in a dynamic physiological environment, the exponential comparative trend (a 535% stress increase) remains a robust and critical baseline for understanding the pathogenesis of unicompartmental osteoarthritis and for preoperative surgical planning.

The present research successfully quantified, via a high-fidelity finite element model, the mechanical severity induced by an 18° genu valgum deformity. The results obtained allow for the establishment of three determining conclusions for clinical biomechanics:

- Critical Stress Disparity: It was demonstrated that the alteration of the mechanical axis does not generate a linear load increase, but rather an exponential one. The pathological knee registered a maximum stress of 17.54 MPa in the lateral compartment, in contrast to the 2.76 MPa observed in healthy morphology. This 535% increase confirms that severe deformities greater than 15° subject the menisci and subchondral bone to stresses that exceed the physiological fatigue limits of the biomaterials, irreversibly accelerating joint degeneration.
- Insufficiency of Conservative Treatments in Severe Grades: Numerical data suggest that, for angular deviations of this magnitude (18°), mechanical overload is structural and cannot be effectively compensated by muscular rehabilitation alone. The local stress of 17.54 MPa supports the need for corrective surgical interventions (such as osteotomies or arthroplasties) to realign the load axis before irreversible meniscal collapse occurs, as the load exceeds the biological tissue’s absorption capacity.
- Validation of Biomodelling as a Preoperative Tool: The implemented methodology, based on DICOM image segmentation and quadratic 20-node meshing, proved to be a robust predictive tool. This approach allows surgeons to visualize and quantify patient-specific stress zones prior to intervention, facilitating the planning of precise bone cuts or the design of custom implants that optimize load redistribution and prolong the lifespan of the natural or prosthetic joint.

## Data Availability

The original contributions presented in the study are included in the article/supplementary material, further inquiries can be directed to the corresponding author.
